# Unspoken words: decoding the dialog between type 2 innate lymphoid cells and T cells

**DOI:** 10.3389/fimmu.2025.1677358

**Published:** 2025-10-06

**Authors:** Zahra Jamila Ikra, Qiutong Huang, Huiyang Yu, Gabrielle T. Belz, Craig N. Jenne, Nicolas Jacquelot

**Affiliations:** ^1^ Department of Biochemistry and Molecular Biology, Cumming School of Medicine, University of Calgary, Calgary, AB, Canada; ^2^ Department of Microbiology, Immunology and Infectious Diseases, Cumming School of Medicine, University of Calgary, Calgary, AB, Canada; ^3^ Arnie Charbonneau Cancer Institute, University of Calgary, Calgary, AB, Canada; ^4^ Frazer Institute, The University of Queensland, Woolloongabba, QLD, Australia; ^5^ Calvin, Phoebe, and Joan Snyder Institute for Chronic Diseases, University of Calgary, Calgary, AB, Canada; ^6^ Alberta Children’s Hospital Research Institute, University of Calgary, Calgary, AB, Canada

**Keywords:** innate lymphoid cells, ILC2, T cells, adaptive immunity, antigen presentation

## Abstract

Type 2 innate lymphoid cells (ILC2s) are critical mediators of type 2 immunity that play non-redundant context-dependent modulatory functions. Primarily associated with responses against helminths and allergens via the activation of a potent epithelial-ILC2 axis, a growing body of evidence also suggests that a crosstalk between ILC2 and T cells is equally important in maintaining tissue homeostasis. In barrier tissues and secondary lymphoid organs, ILC2s co-localize with T cells, forming hubs where bi-directional signals are exchanged. Here, we describe the diversity of functional interactions between ILC2s and T cells, detailing known contact-dependent and -independent mechanisms, including a relatively new and still poorly defined antigen-presenting function during inflammation. Understanding these complex interactions is necessary to fully elucidate how this specific crosstalk helps maintain tissue homeostasis and regulate inflammatory responses. Identifying the spatial and temporal specificities of these interactions will certainly open new avenues for future targeting of this axis to improve immune-mediated host protection.

## 1 Introduction

The immune system is complex and encompasses a wide range of cell types and tissue structures, that together, have been evolutionarily selected to provide optimal protection against pathogen infections and damage to the host (injury, cancer, etc.). Often dichotomized into innate and adaptive compartments based on antigen-specificity and the capacity to generate a memory response, a finely tuned interplay between immune cells is required to mount an effective immune response. Successful cooperation between innate and adaptive immune cell subsets ultimately dictates the immune outcome. Naïve adaptive lymphocytes, such as T and B cells, patrol the body searching for their cognate antigen. Conversely, innate immune cells, often considered as the first line of defense, preferentially reside in tissues, ideally positioned to detect any danger signals (1). They form a dense network of cells that constantly sense their environment to detect potential threats. Once they identify a danger, these tissue-resident immune cells collaborate to amplify the immune response and orchestrate protective immunity, which culminate in antigen-specific T cell activation and B cell-derived antibody production. Among these tissue-resident innate immune cells, professional antigen presenting cells (APCs) such as dendritic cells (DCs), which upon capture of and activation from a pathogen, migrate to the regional tissue-draining lymph node where they present antigen to naïve T cells (2). Activation of T cells is a key step in fighting off pathogens and forming long term protection by creating immunological memory.

Besides professional APCs, innate lymphoid cells (ILCs) emerge as a family of cells that have the capacity to modulate the adaptive immune response through contact-dependent and -independent mechanisms (3–5). These ILCs originate from the common lymphoid progenitor (CLP) and share functional characteristics with CD4^+^ and CD8^+^ T lymphocytes, but do not express antigen-specific receptors (6). Despite similarities between ILCs and T cells, recent studies have shown that they exhibit non-redundant functions (7–11), though specificities may exist such as in the context of worm infectious models (12). ILCs express surface and intracellular receptors to sense their surroundings, enabling them to get activated by signals they receive from their environment. These signals include a wide variety of inflammatory mediators, danger signals, stress ligands expressed by cellular neighbors, neuropeptides, hormones, neurotransmitters, diet-derived molecules as well as metabolites which trigger an ILC-mediated response that impacts inflammation and its resolution. This family of cells is classified into three main groups and further subdivided into five subsets based on their developmental trajectories, transcription factors and cytokines they express (13). Group 1 ILCs (ILC1s) and NK cells parallel CD4^+^ T helper (Th) 1 cells and CD8^+^ T cells, respectively, and confer immunity against intracellular infections and tumors. ILC1s express the transcription factor T-box expressed in T cells (T-bet) and produce type 1 cytokines such as TNFα and IFN-γ (13, 14). Group 2 ILCs (ILC2s) are analogous to Th2 cells and are regulated by the transcription factor GATA Binding Protein 3 (GATA3). ILC2s are involved in immune responses to allergens and parasitic infections, through the production of type 2 cytokines like Interleukin (IL)-5 and IL-13 (15). Group 3 ILCs (ILC3s), including lymphoid tissue-inducer (LTi) cells, share similarities with Th17/22 cells and respond to extracellular fungi and bacteria to fight off pathogens and drive the formation of secondary lymphoid organs (16).

**Figure 1:**
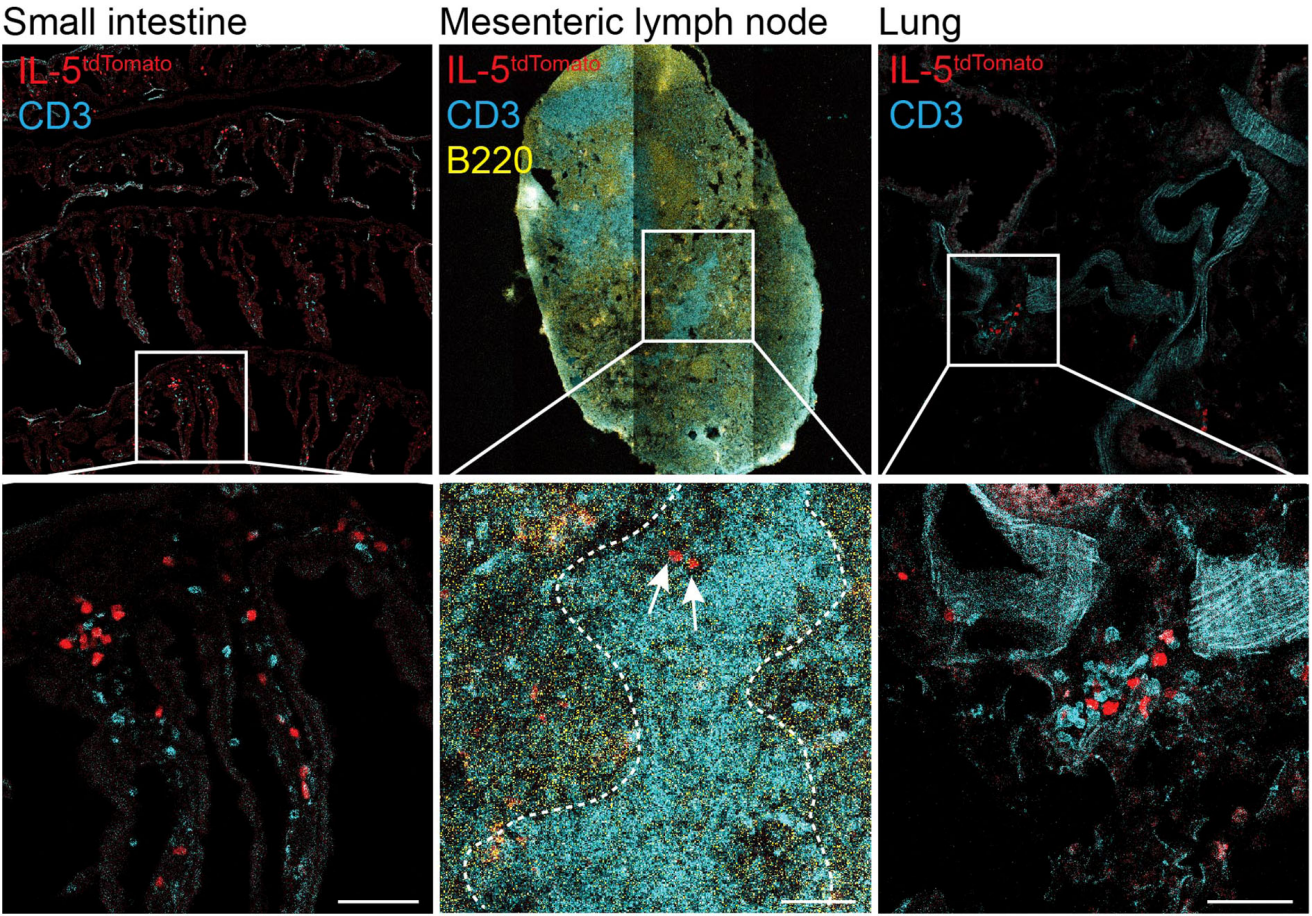
ILC2 and T cells co-localize in the small intestine, mesenteric lymph nodes and lungs. Confocal images showing IL-5Tom^+^ cells (red), CD3^+^ T cells (cyan) and B220^+^ B cells (yellow) in the small intestine, mesenteric lymph node and lungs of IL-5^tdTomato^ mice. Top row shows tiled images. Dotted lines on mesenteric lymph node indicate T cell zone. Arrows indicate IL-5Tom^+^ cells. Images were obtained at 20X magnifications (scale bar represents 50μm). The *IL-5^tdtomiCre/+^* (*B6(C)-Il5tm1.1(icre)Lky/J*; strain 030926) mice were purchased from Jackson Laboratory and have been previously described (18). Mice were bred and maintained in specific pathogen free conditions at the University of Calgary. Experimental procedures were approved by the University of Calgary Animal Ethics Committee (#AC23_0054 and #AC23_0003).

Over the past decade, seminal studies have shown that ILCs play a critical role in regulating adaptive immunity, shaping diverse T cell responses in the periphery, with intestinal ILC3s taking the center stage (5, 17). Increasing evidence also suggest ILC2s as key modulators of adaptive immune responses, having the capacity to prime and activate both CD4^+^ and CD8^+^ T cells. ILC2s and T cells share common anatomical sites and co-localize in both lymphoid and non-lymphoid organs, favoring direct interactions (**Figure 1**). In addition, ILC2-derived cytokines, chemokines, and soluble factors, not only influence the activation of nearby T cells, but also act distally to modulate the activity and function of other cells, such as eosinophils, which culminate in influencing T cell migration and effector function (18, 19). Communication is a two-way exchange of signals and as such, T cells reciprocally influence ILC2 functions through the expression of surface molecules and secretion of soluble factors. In this review, we discuss ILC2 and T cell positioning in tissues, including secondary lymphoid organs, and how this proximity enables an ILC2-T cell dialogue shaping immunological responses and tissue protection. We will elaborate on cell-cell membrane interactions and soluble mediators that participate in the ILC2-T cell crosstalk, and discuss ILC2s capacity to uptake, process and present antigens to prime and activate T cells. Despite the basic understanding of the interplay between ILC2s and adaptive immune cells, it remains a challenge to develop tailored targeting strategies to modulate this ILC2-T cell axis, potentially in a tissue-specific manner, to enable fine tuning of local inflammation.

## 2 Discovery, phenotype, and function of type 2 innate lymphoid cells

ILC2s were first discovered in 2006 (20) and subsequently redescribed in 2010 (15) as a non-T cell, non-B cell subset, capable of producing type 2 cytokines in response to the type 2 inducers IL-25 and IL-33, and play a central role in the fight against helminth infections like *Nippostrongylus brasiliensis (* 15, 20–22). Based on their phenotype and location, two main subsets of ILC2s have been described thus far. At steady-state, in lung and adipose tissues, ‘Natural’ ILC2s (nILC2s) preferentially respond to interleukin (IL)-33 via high expression of its corresponding receptor ST2. In contrast, ‘Inflammatory’ ILC2s (iILC2s (23)) in the small intestine express low levels of ST2 and preferentially respond to IL-25 stimulation owing to the expression of the IL-25 receptor, IL-17RB. Furthermore, upon activation, ILC2s can downregulate CD127 and CD90 and upregulate KLRG1 expression. CD25^low^ST2^low/-^KLRG1^hi^ iILC2s are also observed in mesenteric lymph nodes, spleen, liver, and lungs upon IL-25 challenge or helminth infection models like *N. brasiliensis* and *Tritrichomonas musculis (* 24, 25). Therefore, ILC2 subsets are not single, homogeneous entities, but rather are composed of a diverse array of cells harboring distinct phenotypes associated with specific cell states. At steady-state, ILC2s are mainly located at the body barrier surfaces such as lung, skin and small intestine, but can also be found in other tissues such as peritoneum, liver, bone marrow, and adipose tissue as well as in secondary lymphoid organs (26) (**Figure 1**). ILC2 identification relies on the absence of lineage markers expression (mouse: CD3, TCRαβ, TCRγδ, CD19, B220, CD11c, CD11b, F4/80, Gr-1; Human: CD3, TCRαβ, TCRγδ, CD19, CD20, CD34, CD123, CD11c, CD303, FcεR1) together with the expression of CD90, CD127 and high expression of GATA3. Other widely used markers to identify ILC2s in tissues include Sca-1, KLRG1, IL-17RB, ST2, and CD25, although variation in their level of expression exists depending on which tissue ILC2s reside in and their level of activation (15, 27). In humans, ILC2s characterized as lineage^-^CD56^-^CD127^+^CRTH2^+^ have been identified in circulation and within the human adipose tissue, lung, small intestine, mediastinal and mesenteric lymph nodes and colon (28).

ILC2s are widely recognized as important mediators for type 2 immunity. In the small intestine, ILC2s provide immunity against helminths and extracellular pathogens (8, 11, 26, 29, 30), while in the lung, ILC2s are involved in amplifying allergen-driven immune responses and tissue repair. Both lung and intestinal ILC2s are activated by canonical type 2 factors like IL-2, IL-25, IL-33 and thymic stromal lymphopoietin (TSLP). Despite low expression of some receptors for these cytokines in specific anatomical sites, ILC2s have demonstrated the capacity to respond to these stimulatory signals. Upon stimulation, ILC2s express the tissue repair molecule amphiregulin and the cytokines IL-4, IL-5, IL-9, IL-13, and Granulocyte-macrophage colony stimulating factor (GM-CSF) (31, 32). These ILC2-derived cytokines commonly promote eosinophilia, macrophage mobilization and suppression of type 1 immune responses, depending on ILC2 localization, their microenvironment and the inflammatory context.

## 3 Type 2 innate lymphoid cells – tissue-resident cells but not immobile

Type 2 ILCs can develop and adapt tissue-specific morphologies and functions. Despite being primarily tissue-resident, recent studies have clearly shown that ILC2s are not static. Using photoconvertible Kaede mice to differentiate between migratory and resident cells within tissues, Mackley et al. (33) observed that, at steady-state, intestinal ILC2s are mobile and can migrate from the intestine to the mesenteric lymph nodes. Since this finding, many studies have reported the capacity of skin, lung and intestinal resident ILC2s, among others, to migrate to tissue draining lymph nodes or to distant organs (34–36). The trafficking potential of these ILC2s is enhanced following activation and cells can migrate, not only to the draining lymph node, but also to distant sites, infiltrating inflamed tissues where they respond to local cues. For example, inflammatory KLRG1^+^ ILC2s (iILC2s) induced by IL-25, or a *N. brasiliensis* infection, expressed the sphingosine 1-phosphate (S1P) receptor which induced the migration of iILC2s from the gut to the lungs through the lymph (37). In a *Tritrichomonas musculis* model, activated ILC2s residing in the small intestine migrate to the lungs in a S1P receptor 4 (S1PR4) dependent manner, where they are sustained by IL-2 from T cells, and inducible T cell costimulatory ligand (ICOSL) from B cells (25). These observations highlight the capacity of ILC2s to be mobilized when needed and support inflammatory responses at distant sites when required.

### 3.1 Skin ILC2s

The immunological composition of the skin is very specific and is enriched in innate and innate-like immune cells, like ILC2s. Skin ILC2s, characterized by high expression of IL-18Rα, influence DC homeostasis (38, 39), eosinophil recruitment and dermal skin-resident macrophages (40), and contribute to tissue homeostasis by regulating the skin microbiota (41, 42). At steady-state, skin-resident ILC2s do not migrate from the skin to the skin-draining lymph node (43, 44). However, single-cell RNA sequencing analyses of ILC2s isolated from the skin and skin-draining lymph nodes of mouse model with atopic dermatitis (IL-33 overexpression; IL-33tg mice) (45), revealed the existence of two distinct clusters of ILC2s: one corresponding to cells that preferentially reside in tissues and one composed of circulating ILC2s. Following photoconversion of cells in the skin of IL-33tg mice, a small proportion of photoconverted ILC2s were found in the draining lymph node, indicating that during inflammation, ILC2s can migrate from the skin to the closest lymph node (43). However, this observation might be tied to the IL-33tg mice as topical application of MC903 to induce skin inflammation failed to mobilize skin-resident ILC2s and induce their migration from the skin to the draining lymph node, despite increased ILC2 numbers in the latter (44).

### 3.2 Lung ILC2s

The lungs are home to tissue resident cells, including ILC2s, that participate in the maintenance of tissue homeostasis. At steady-state, lung ILC2s localize in specific niches, close to adventitial stromal cells (ASCs) that express IL-33 and TSLP, two key cytokines involved in maintaining ILC2 homeostasis and effector function. Depletion of ASCs during helminth infection impaired ILC2 accumulation and lung type 2 immune responses (46). In a separate study, intranasal administration of IL-33 or IL-25 resulted in an increased expression of CXC chemokine ligand 16 (CXCL16) in the lung parenchyma, with IL-33 inducing a more immediate and long-lasting expression of CXCL16, as compared to IL-25. Intranasal administration of IL-33 and CXCL16 into the airways induced the trafficking of CXCR6^+^ ILC2s from the peripheral circulation into the lungs. CXCL16 blockade in mice challenged with IL-33 resulted in decreased migration of ILC2s into the lungs (47). This revealed a crucial role for IL-33-mediated CXCL16 overexpression in promoting an ILC2-driven type 2 immune response in this model of asthma.

### 3.3 Intestinal ILC2s

To maintain peripheral tolerance to commensal microbiota, and detect and eliminate potential harmful pathogens, the intestine contains up to 70% of the body’s immune cells (48). Amongst these mucosal immune cells, ILC2s localize in the lamina propria, immediately underlying the epithelial lining, maintain intestinal homeostasis and protect against pathogens and infections, particularly helminths. The interplay between tuft cells, a specialized epithelial cell type, and ILC2s allows for effective expulsion of helminths and elimination of the parasites from the intestine. In tuft cell-deficient mice, or in the absence of ILC2s, increased worm burden was observed (49), highlighting the key role of the ILC2-tuft cell axis in protecting the host against helminth infection. Inflammatory intestinal ILC2s also have the capacity to migrate to distant organs where they can respond to local inflammatory cues (49). Ricardo-Gonzalez and colleagues (50) studied the kinetics of ILC2 translocation between blood and the small intestine, and the resulting phenotypic variation during infection with *N. brasiliensis*. Five days after infection, an increase in the frequency of ILC2s phenotypically similar to those resident in the lamina propria which express both KLRG1 and IL-17RB but not ST2 or arginase 1 (Arg1), was observed in the blood. By 12 days after infection, these cells were replaced by ILC2s phenotypically similar to those in the lungs which express KLRG1, ST2 and Arg1 but had reduced IL-17RB expression. Fate-mapping analysis, a technique applied to track and study the developmental origin of cells using fluorescent dyes that mark cells over their lifespan, revealed the existence of two waves, a ‘gut wave’ and then a ‘lung wave’ of ILC2s that circulate in the blood. In line with their tissue of origin, gut-derived ILC2s were dependent on IL-25 signaling, whereas lung-derived ILC2s relied on IL-33 signaling. Treating mice with an antiparasitic drug that abrogates the gut phase of the helminth infection resulted in a marked decrease in the number of ILC2s during the “gut wave” in the blood on day 5 after infection, with no change in the number of ILC2s during the “lung wave” on day 12 post infection. A similar effect was observed for “lung wave” ILC2s when the lung affecting phase of the helminth’s life cycle is circumvented (50). Intestinal ILC2s can respond to IL-33 stimulation despite low ST2 expression. In pancreatic tumors, IL-33 activated ILC2s from the gut migrate to tumors and form tertiary lymphoid structures (TLS) that are correlated to improved prognosis (36). Altogether, migration of ILC2s between the intestine and distant tissues is a dynamic process necessary to optimally protect the host against pathogens.

Collectively, these studies show the importance of ILC2s in influencing tissue homeostasis and orchestrating immune responses during inflammation. Spatial mapping of ILC2s has revealed ILC2s are located in specific niches enabling them to receive and respond to various signals and interact with both immune and non-immune cells, ultimately shaping their effector function.

## 4 Type 2 innate lymphoid cell – T cell co-localization in tissues

ILC2s are found in all tissues though their proportion and localization may significantly differ according to the tissue of their residency (24, 51, 52). For instance, in the lung, brain, gonadal adipose tissue, liver, and kidney, ILC2s are localized in the perivascular adventitial cuff structures (PACS) (46). Tissue distribution of other ILC subsets have been comprehensively reported elsewhere (24, 51, 52) and is not a major focus of discussion below. Rather, we concentrate on the ILC2–T cell co-localization and how this proximity enables contact-dependent and -independent interactions, shaping the effector functions of both subsets.

### 4.1 Lymph nodes

Lymph nodes are key structures for both B and T cell activation and the generation of adaptive immune responses. In lymph nodes, ILC2s localize in inter-follicular regions, zones of T cell-B cell interactions (**Figure 1**). ILC2s interact with T cells (33, 53), potentially influencing T cell and B cell effector function and fate (54). During helminth infection, increased interactions between ILC2s and T cells have been detected at the mucosal barrier whereas, in lymph nodes, reduced duration of interaction between T cells and ILC2s was observed (53). The analysis of T cell deficient mice (*Rag^-/-^*, *Zap70^-/-^*, *Tcrα^-/-^*, and *Cd3ϵ^-/-^*) has revealed an accumulation of ILC2s in mesenteric (33, 55) and peripheral (55, 56) lymph nodes compared to wild-type animals. Since T cells and ILCs express similar cytokine receptors and rely on similar pathways for their development, survival and effector functions, they compete for access to the same signals locally (57) and as such it is perhaps not entirely surprising that in the absence of T cells, increased numbers of ILC2s are found in tissues. Reconstitution of the T cell pool in *Cd3ϵ^-/-^* mice lead to a downregulation of *Il33* expression in stromal cells culminating in reduced ILC2 expansion to levels similar to those found in wild-type animals (53). Additionally, two receptors namely signaling lymphocyte-activating molecule (SLAM) family receptors 3 (SLAMF3) and 5 (SLAMF5) expressed on T and B cells inhibit the growth and maintenance of ILC2s in the mesenteric lymph node, in an IL-7 dependent manner (58). These findings suggest a role for T cells in regulating ILC2 homeostasis in lymph nodes and potentially in other tissues through local modulation of IL-33 expression.

### 4.2 Skin

In the skin, ILC2s reside in the dermis and subcutis layers, with specific phenotypes pertaining to their respective function (59). ILC2 interaction with T cells in the skin has not been extensively studied. It has been reported that retinoid-related orphan receptor (ROR) expression in regulatory CD4^+^ T cells (T_regs_) is required for repressing ILC2-driven allergic skin inflammation (60). During skin inflammation, in a model of atopic dermatitis, Malhotra et al. showed that the deletion of *Rora* in T_regs_, alleviated T_reg_-induced ILC2 suppression in a contact dependent manner, enhancing ILC2-derived IL-5 expression, eosinophilia, and a type 2 immune response (60). Following activation through keratinocyte-mediated IL-25 expression, ILC2-derived IL-13 expression aided in the recruitment of CD4^+^ T cells into the inflamed skin, via the expression of the chemokines CCL17 and CCL22 (61).

### 4.3 Lungs

Tertiary lymphoid structures called inducible bronchus associate lymphoid tissues (iBALT) are formed in the lungs following allergen or viral exposure. These structures house antigen-specific B and T cells along with DCs leading to localized inflammation (62). A recent study showed that upon rag weed pollen (RWP) exposure, ILC2s colocalize with T and B cells in iBALTs near pulmonary lymphatic vessels (7). Lining the airways are peribronchial spaces that maintain ILC2s and CD4^+^ T cells in close proximity (7). Using a T cell-specific IL-4 and IL-13 knockout mouse model, Symowski and Voehringer showed that Th2-derived IL-4 and IL-13 expression are vital for ILC2 expansion in the lungs following *N. brasiliensis* infection (63). During the infection, STAT6 signaling in ILC2s not only induced ILC2 proliferation and Major Histocompatibility Complex (MHC) class II (MHC II) expression, but also promoted migration of ILC2s to the lungs (63). In a house dust mite-induced allergic reaction, ILC2 proliferation and activation is dependent on Th2 activation and the expression of T cell-derived cytokines IL-2 and IL-21 (64). In contrast, ILC2-derived IL-13 is required for the priming of naïve T cells and their differentiation into Th2 cells in papain-induced lung asthma model (65). During the course of *N. brasiliensis* infection, a crosstalk between ILC2s and CD4^+^ T cells in the lungs, involving the interaction of PD-1 expressed on T cells with its ligand PD-L1 expressed on ILC2s, sustains a robust type 2 immune response, resulting in improved parasite expulsion (66).

### 4.4 Intestine

Using intravital imaging techniques, Lok et al. tracked ILC2s in the small intestine and Peyer’s patches and found that ILC2s co-localized with T cells in the mucosa of the small intestine at steady-state, similar to our observations (**Figure 1**). During helminth infection, a proportion of mucosal ILC2s interact with neighboring T cells, maintaining prolonged cell-cell interactions, to an extent similar to that required for antigen presentation (67).

Together, the mapping of ILC2s and T cells in tissue barriers and lymph nodes has revealed a close spatial proximity between these two cell types, suggesting robust ILC2-T cell crosstalk that influence each other’s subsets and effector functions. While our understanding of this ILC2-T cell crosstalk remains limited, recent studies have revealed reciprocal ILC2-T cell interactions that involve both contact-dependent and -independent mechanisms, ultimately shaping the breadth and magnitude of the immune response during inflammation.

## 5 ILC2 - T cell crosstalk

A well-coordinated immune response requires immune cells to interact with each other. Direct interactions involve physical proximity and cell-cell contact that imply the engagement of cell surface receptors and ligands, and the exchange of soluble factors such as chemokines and cytokines. Indirect interactions typically occur between spatially distant cells and involve the release of soluble factors which act on target cells that express the corresponding receptors. Effective immune responses require optimal communication between immune cells. These notably include interactions that occur between ILC2s and T cells.

### 5.1 Direct modulation of T cell function

#### 5.1.1 Antigen presentation and CD4^+^ and CD8^+^ T cell priming

**Figure 2:**
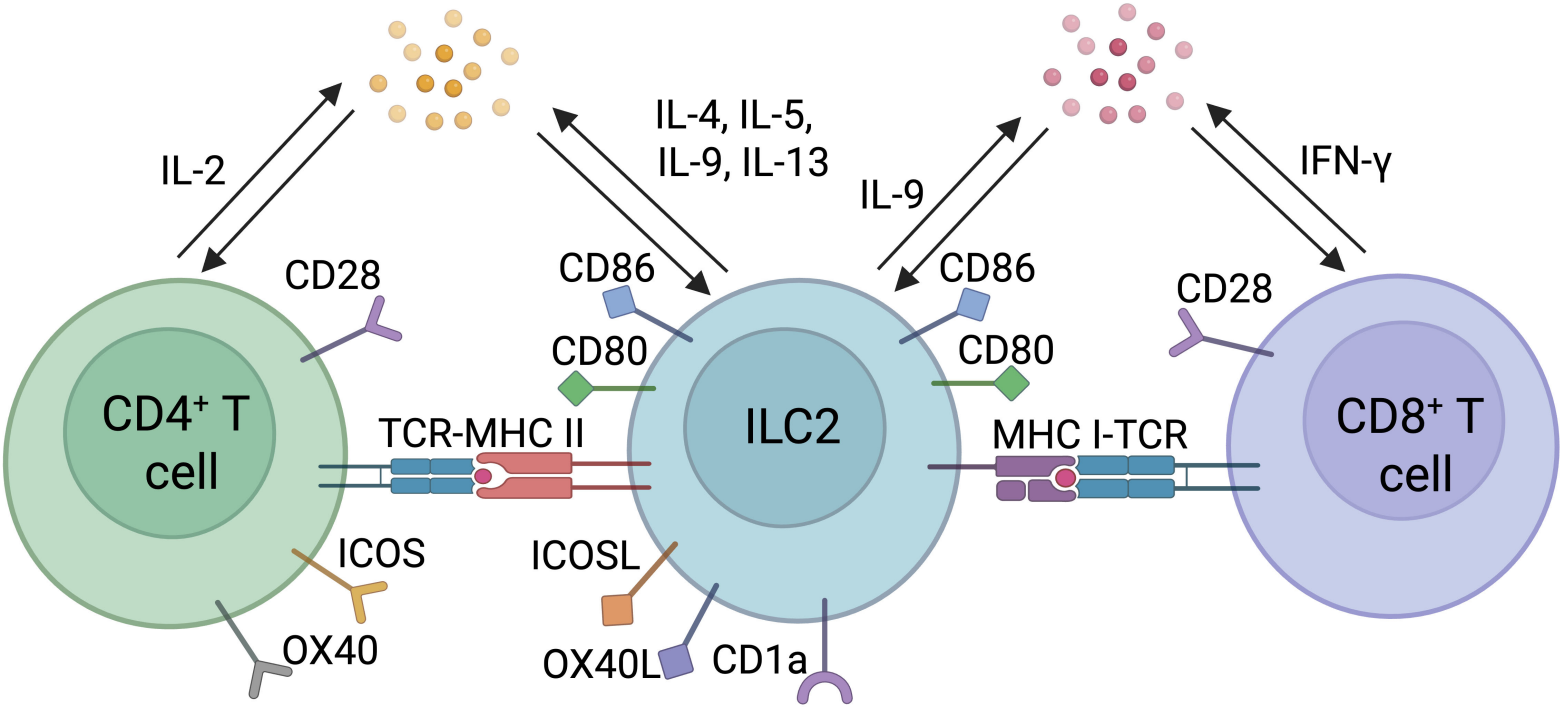
Direct interactions between ILC2s and T cells. Direct interactions between ILC2s and CD4^+^ or CD8^+^ T cells include antigen presentation through engagement of T cell receptor (TCR) and Major Histocompatibility Complex Type I or II (MHC I or II) loaded with peptide, co-stimulatory molecules, and polarizing cytokines.

Professional APCs such as DCs process and present antigens to naïve CD4^+^ and CD8^+^ T cells ultimately priming and activating these key lymphocyte subsets. This process involves the recognition of a specific peptide- MHC class I (MHC I) or II (MHC II) complex by the T cell receptor (TCR), the binding to co-stimulatory molecules, and the production of polarizing cytokines. MHC I is expressed on all nucleated cells and have the ability to present endogenous antigens to CD8^+^ T cells, allowing the detection and elimination of potentially infected or mutated cells. Besides DCs, other professional and non-professional antigen presenting cells have been described, having the capacity to prime naïve T cells or influence T cell effector responses in an antigen-specific manner. Among them, B cells and macrophages are well known antigen presenting cells (68, 69). While the expression of MHC II is more restricted and remains limited to specific immune cell subsets, other cells can express or acquire peptide-loaded MHC II molecules, for example, through trogocytosis (70), that confers antigen-presenting functions. These MHC II^+^ cells include B cells, macrophages and other myeloid cell subsets, stromal cells and ILCs (2, 5, 68). Often referred as to non-professional APCs as opposed to dendritic cells which are considered as professional APCs, increasing evidence suggest that they can shape the breadth and depth of the T cell response, particularly CD4^+^ T cells. In the intestine, the recent discovery of RORγt^+^ APCs that direct the differentiation of CD4^+^ T cells into microbiota-specific peripheral Tregs that express both Foxp3 and RORγt highlights the role of non-DCs in CD4^+^ T cell priming and differentiation. MHC II^+^ cells present extracellular antigens to naïve or already primed CD4^+^ T cells enabling controlled polarization of the immune response. Recent findings indicate that ILC2s exhibit all the required features for antigen presentation with the expression of the necessary antigen processing machinery along with the expression of MHC I, MHC II and other MHC proteins such as CD1a, enabling them to uptake, process and present antigens to CD8^+^ and CD4^+^ T cells (71, 72) (**Figure 2**). In addition, ILC2s also express co-stimulatory molecules such as inducible T cell costimulator (ICOS) and its ligand, ICOS-L, as well as OX40L, CD80, CD86, all of which having distinct but complementary roles in inducing and amplifying T cell-mediated immune responses (72).

##### 5.1.1.1 MHC II expression and CD4^+^ T cell priming

ILC2s express MHC II molecules enabling them to present antigens to CD4^+^ T cells. At steady-state, lung ILC2s express high levels of MHC II and its blockade in *in vitro* co-culture system with antigen-specific T cells results in reduced cytokine production by ILC2s. Ovalbumin-pulsed ILC2s induced CD4^+^ T cell proliferation, an effect that was lost following co-culturing ILC2s and OT-II cells with a neutralizing anti-MHC II antibody. Furthermore, IL-2 produced by CD4^+^ T cells directly amplifies ILC2 cytokine expression (73), leading to the expression of high levels of the type 2 cytokines IL-4, IL-5, IL-9 and IL-13. This effect was suppressed by the addition of MHC II neutralizing antibodies, implicating a direct involvement of MHC II and antigen presentation in promoting ILC2-derived cytokine expression. Not only lung ILC2s but also ILC2s from the small intestine and lymphoid tissues express MHC-II (74). MHC II expression and subsequent activation of CD4^+^ T cells results in an IL-13-dependent Th2 response and clearance of helminth infection. The expression of MHC II is conserved between mouse and human ILC2s, highlighting the importance of this pathway in ILC2 biology and type 2 immune responses (72). It remains to ascertain whether MHC II^+^ ILC2s are capable of direct priming of naïve CD4^+^ T cells, not only *in vitro*, but also *in vivo*. It is also possible that rather than priming *de novo* naïve T cells, ILC2s modulate the effector functions of already primed effector T cells in a TCR cognate manner through peptide-MHC II interaction and expression of co-stimulatory molecules as an evolutionary mechanism to amplify T cell response at the site of inflammation.

##### 5.1.1.2 MHC I expression and CD8^+^ T cell priming

Cross-presentation of extracellular antigens to CD8^+^ T cells is a process that remains restricted to type 1 conventional dendritic cells. Intriguingly, in a lung cancer model, Wen and colleagues (75) recently described the ILC2s capacity to cross-present antigen to CD8^+^ T cells. They found that IL-33-activated ILC2s expressed genes encoding for MHC I machinery and costimulatory molecules. The gene signature of IL-33-activated ILC2s was similar to the gene signature of DCs activated by LPS stimulation and the expression of many of the genes related to antigen presentation has been confirmed at the protein level. Importantly, IL-33-activated ILC2s have also been shown to have the capacity to phagocytose antigenic proteins (76), highlighting the cross-presentation potential of extracellular antigens to CD8^+^ T cell. Using the widely employed OT-I cells, a model used to study specific CD8^+^ T cell responses to ovalbumin, the co-culture of ILC2s and CD8^+^ T cells in the presence of the full-length ovalbumin protein or an ovalbumin-derived peptide induced T cell proliferation and activation. In addition, co-culture of lung ILC2s with naïve T cells resulted in increased proliferation, effector function, and cytotoxic potential of CD8^+^ T cells in a contact-dependent manner (76). Activated ILC2s in the lung also express MHC I, a phenotype that is more pronounced following co-culture with CD8^+^ T cells. The capacity of lung ILC2s to cross-present antigens, prime, and activate CD8^+^ T cells increases CD8^+^ T cell cytotoxicity in the tumor microenvironment and reduces tumor burden (77).

##### 5.1.1.3 Expression of costimulatory molecules

The expression of either MHC I or MHC II molecules along with peptide presentation is not sufficient to effectively prime and activate T cells. Full T cell activation requires additional signals often referred to as second and third signals. ILC2s express a wide range of costimulatory molecules which are able to bind to their corresponding ligands or receptors expressed on T cells. Similar to DCs, ILC2s express CD80 and CD86, both of which bind to CD28 expressed on naïve T cells. ILC2s also express both ICOS and its ligand, ICOS-L (67, 72, 78). ICOS binds to ICOS-L, which induces the production of IL-4 and IL-13 by CD4^+^ Th2 cells (79, 80). ILC2s also express OX40 ligand (OX40L), a co-stimulatory molecule that has been involved in the activation of OX40^+^ T cells and their differentiation into Th2 cells during *N. brasiliensis* infection, leading to efficient helminth expulsion (81). In a type 2 inflamed niche, OX40L expression by ILC2s associated with ILC2-derived CCL1 secretion mediate the recruitment and accumulation of GATA3^high^ T_regs_ expressing OX40 and CCR8. This ILC2-T_reg_ interaction directly impairs the expansion of effector Th2 cells by limiting OX40L bioavailability thus preventing excessive type 2 inflammation which might result, if uncontrolled, in irreversible tissue damage (82). Butyrophilin 2a2 (Btn2a2) is another costimulatory molecule, previously defined on T cells, that is expressed on ILC2s at steady-state (83). Using both *in vitro* and *in vivo* models, Frech et al. found that the expression of Btn2a2 on ILC2s impaired CD4^+^ T cell response against helminth infections while the adoptive transfer of Btn2a2-deficient ILC2s along with antigen-specific CD4^+^ T cells into Ragyc^-/-^ mice resulted in increased frequency of CD4^+^ T cells as well as IL-4 and IL-13 expression in CD4^+^ T cells and ILC2s (83).

#### 5.1.2 Modulation of T cell function through soluble factors expressions

##### 5.1.2.1 Cytokines

Cytokines produced by ILC2s after activation influence T cell activity and effector function. In an ILC2-CD4^+^ T cell co-culture system, ILC2-derived IL-4 expression promotes the production of IL-5 and IL-13 by CD4^+^ T cells (84). In a papain-induced lung inflammatory model, the production of IL-13 by ILC2s promotes CD4^+^ T cell activation and Th2 polarization (65) while in an HDM-mediated airway inflammatory model, the activation of T cells precedes that of ILC2s and T cell-derived IL-2 and IL-21 expression promote ILC2 activation (64). Wan and colleagues (85) found that the secretion of IL-9 by ILC2s *in vitro* activate CD8^+^ T cells favoring T cell-mediated killing of CT26 cancer cells. *In vivo*, the neutralization of IL-9 using a blocking antibody resulted in increased CT26 tumor growth. While blocking ILC2s in nude mice that are deficient for T cells has no effect on tumor burden, adoptive T cell transfer into ILC2-depleted mice resulted in increased tumor burden compared to ILC2-sufficient animals. These observations suggest a direct crosstalk between ILC2s and T cells requiring IL-9 signaling for T cell-mediated cancer cell killing (85). In addition, IL-2 production by activated CD4^+^ T cells modulates ILC2-derived IL-5, IL-9, and IL-13 expression (73, 86). In contrast, the presence of type 1 cytokines such as IFN-*y* and type 1 IFN inhibits ILC2 activity (87). In the liver, IFN-*y* directly counteracts IL-33-mediated ILC2 proliferation and IL-5 and IL-13 production, whereas blocking IFN-*y* restores the frequency of ILC2s which express IL-5 and IL-13 (78). Amphiregulin (AREG) is a soluble factor that was shown to modulate the crosstalk between ILC2s and T cells. IL-33 stimulated ILC2s express AREG (88) which, on binding to the epidermal growth factor receptor (EGFR) expressed on T_regs_, induces the flip of the integrin α_V_ to the outer membrane leading to the release of active TGF-β from latent complexes that accumulate in the local environment (89, 90), thereby increasing T_reg_ suppressive functions (87, 91–93). In a lupus nephritis model, ILC2-derived AREG in the kidney suppresses inflammatory T cell signals locally, which prevents T cell activation and disease progression (94). Further investigations are warranted to further identify the relative contribution of ILC2-derived AREG expression to Treg suppressive effector functions, particularly relative to macrophages, mast cells, or other immune cell-derived AREG expression (89, 93, 95), in models of inflammation, infection or carcinogenesis.

### 5.2 Indirect modulation of T cell effector function through the engagement of a third partner

**Figure 3:**
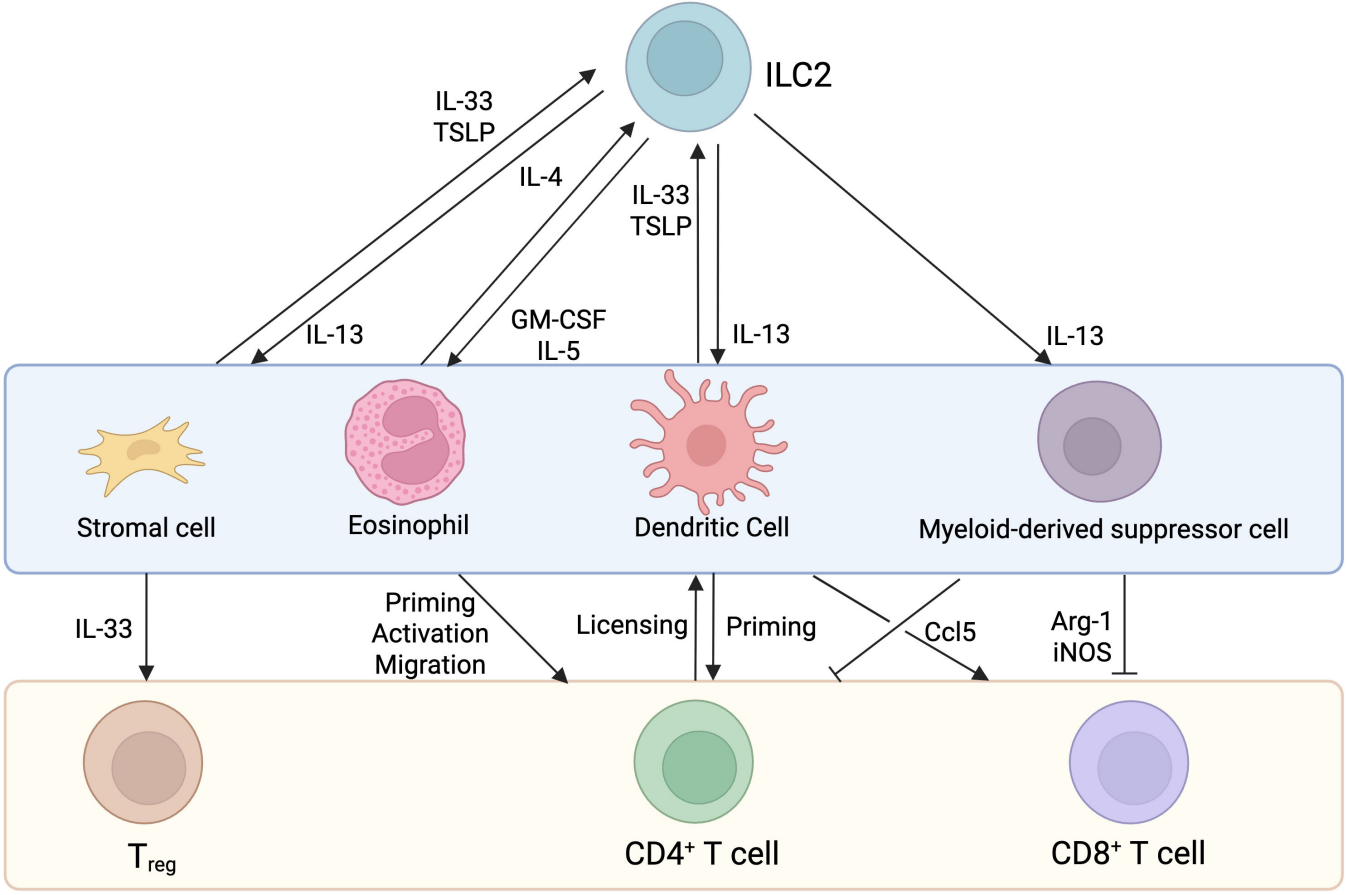
Indirect interactions between ILC2s and T cells. Known indirect interactions involve the expression of cytokines and chemokines that impact stromal cells (in the lungs), eosinophils (in the lungs and skin), dendritic cells (in the lymph nodes, lung, liver and kidney) and myeloid-derived suppressor cells (in tumors) activity, culminating in ILC2 and or T cell activation.

In addition to direct interactions, ILC2s can interact with T cells indirectly through the engagement of other cell types to amplify the immune response (**Figure 3**). ILC2-derived IL-4, IL-5, IL-9, and IL-13 production, not only influence T cell effector functions, but also shape the activity of other immune and non-immune cells such as stromal cells and antigen presenting cells which in turn affect T cell responses.

#### 5.2.1 Stromal cells

Using a reporter mouse model, Dahlgren and colleagues found that ILC2s colocalize with adventitial stromal cells (ASCs), T_regs_, and DCs. These ASCs express *Tslp, Ccl11* and *Il33*, all of which play an important role in type 2 immunity while the latter is a major stimulatory cytokine for ILC2s. IL-33 and TSLP derived from lung ASCs promote the survival and proliferation of ILC2s, both *in vitro* and during helminth infectious models (46).

#### 5.2.2 Myeloid cells

During papain-induced lung inflammation, ILC2-derived IL-13 production promotes the recruitment of antigen presenting DCs to the lymph nodes, where they prime naïve CD4^+^ T cells to induce Th2 cell differentiation and type 2 immunity (96). In lungs, liver, and kidneys, ILC2s are positioned near CD11c^+^ MHC II^+^ dendritic cells, indicating that adventitial perivascular niches are locations for DC-ILC2 interactions (46). Activated DCs produce IL-33, TSLP, and tumor necrosis factor-like ligand 1A that activates ILC2s. Once activated, ILC2s release IL-13 which further promotes DC trafficking into lymph nodes to amplify a type 2 immune response through CD4^+^ T cell priming (97). In cancer, ILC2-derived IL-13 production promotes the recruitment of myeloid-derived suppressor cells (MDSCs) into tumors, inhibiting T cell effector function, culminating in tumor growth and disease progression (98). One of the significant functions of ILC2s is their ability to promote eosinophilia. Eosinophils have been shown to express CCL5, CXCL9, CXCL10 thereby helping in T cell recruitment into the tumor microenvironment (99). PD-1 is expressed by ILC2s and has been shown to drive ILC2 pro-tumorigenic function (100). The blocking of PD-1 promotes ILC2-derived CCL5 expression which recruits DCs, leading to the accumulation of intratumoral antigen-specific T cells (101).

## 6 Concluding remarks

Primarily recognized for their ability to orchestrate type 2 immunity, growing evidence also suggests a key role for ILC2s in influencing type 1 immune responses. Amongst the myriads of signals that influence ILC2 function, the cytokines IL-33 and IL-25 are prime activating factors for ILC2s. At steady-state, ILC2s primarily interact with other tissue resident cells, including some subsets of tissue resident T cells, helping to maintain proper tissue function locally. During inflammation, ILC2s capacity to promote T cell recruitment and activation as well to possibly T cell priming through direct or indirect means, directly contribute to influencing the inflammatory response. Given that ILC2 functions are largely dependent on the local tissue milieu, their interactions with immune and non-immune cells in different organs present an interesting direction to explore as to modulating local inflammation in a tissue-specific manner. In cancer, for instance, an increasing number of studies have highlighted the critical roles that ILC2s play in modulating the tumor microenvironment (102) with new evidence suggesting that ILC2s could directly influence T cell effector function through direct interactions. While further studies are warranted, if proven true, this would open up a new avenue into using ILC2s to influence the anti-tumorigenic capacity of T cells.

## 7 Future perspectives

The capacity of immune cells other than DCs to prime *de novo* naïve T cells remains a matter of debate. While some evidence suggest the induction of T cell responses in the absence of DCs, albeit significantly reduced compared to DC competent animals or individuals, how these T cell responses are mounted remain to be fully elucidated. Not only the breadth but also the quality of this response needs to be evaluated. For instance, in the presence of a bi-allelic mutation in *IRF8* with complete cDC1 deficiency, despite the presence of effector CD8^+^T cells, these T cells have impaired IFN-γ production and CXCR3 expression, suggesting a dramatic effect on type 1 immunity. In addition, the capacity of non-DC immune cells to express MHC II or acquire peptide-loaded MHC II molecules at their surface may contribute to CD4^+^ T cell reactivation or polarization through the expression of key cytokines; for instance, IL-4 expression from mast cells or IL-6 production from B cells promote the differentiation of helper T cells into T_h2_ or T_fh_ cells through the induction of *Gata3* or *Bcl6* expression, respectively (103–105). ILC2s, as discussed above, seem to be equipped with the necessary machinery to directly activate T cells through peptide-MHC-TCR interactions. They also express co-stimulatory molecules and key polarizing cytokines such as IL-9 or IL-13. Despite displaying critical antigen-presenting cell features, including the capacity to migrate to secondary lymphoid organs and co-localize with T cells, formal definitive *in vivo* evidence of their capacity to prime T cells is still missing. Ultimately, a better understanding of these cellular interactions, including the possibility of ILC2s to prime T cells may provide an alternative path to modulate T cell effector functions.

In this review, known interactions between T cells and ILC2s are described, both at steady-state and in context of inflammation and disease. However, further investigations are warranted to properly elucidate the nuances of this interaction. Although increasing evidence suggest that ILC2s function beyond the regulation of type 2 immunity, several key questions remain.

- The relative contribution of ILC2s to CD8^+^ T cell priming, particularly in comparison to professional antigen presenting cells, are not yet fully understood. Future studies will need to evaluate the capacity of ILC2s to prime CD8^+^ T cells in the context of ILC2 deficiency but cDC1 competent animals. The extent and quality of the T cell response will need to be compared, side-by-side, to cDC1-deficient but ILC2-sufficient mice and to fully immunocompetent animals. In this setting, the stimulatory effect of ILC2s on DCs, as previously shown by our group and others, ultimately influencing DC-mediated T cell responses, may be a confounder and will need to be carefully evaluated in a context-dependent manner.
- It remains unclear whether ILC2-mediated T cell priming or re-activation occurs within tissues or requires migration to lymph nodes. As such we propose to block the migration of tissue resident ILC2s to lymph nodes by deleting key chemokine receptors at their surface that would prevent them from trafficking to secondary lymphoid organs. Additionally, the use of photoconvertible mouse models could help define the kinetics of the response and through the use of imaging approaches, to decipher the relative preference of tissue-specific activated ILC2s (photoconverted cells) that have migrated to the secondary lymphoid organs for T cell interactions compared to non-photoconverted cells. Additionally, the use of fluorescent dyes or trackers could also aid in determining the capacity of ILC2s to migrate from the site of inflammation to the draining lymph node and positioning close to T cells.
- It is also not known whether T cell priming by ILC2s is comparable to that mediated by dendritic cells, especially with respect to the signals delivered to T cells. ILC2s have been shown to possess the machinery required for antigen uptake and processing, but the implications of this system for ILC2s and their maturation including signaling pathways involved in this remain to be understood. In this context, IL-33 may play a major role in ILC2 activation and the potential induction of this machinery. The analysis of ST2^-/-^ ILC2s using conditional knock out mice should shed light on the role of this pathway in influencing ILC2-T cell interactions. Additionally, in light of the recent findings from Halim et al., Wen et al. and Kim et al., it has become increasingly important to identify the receptors and pathways involved in antigen uptake, processing, and presentation (72, 75, 77, 81).
- While a growing body of research is describing the non-redundant roles of ILCs and T cells in various contexts, the overall impact of ILC2s on T cell immune responses has not been studied extensively. ILC2s form a bridge between the innate and adaptive immunity, with characteristic features that allow them to play a role in both type 1 and type 2 responses. A disease model like the dengue virus infection, where ILC2s have been implicated in regulatory role in type 1 immunity (106), would be ideal to study ILC2s role in antigen cross-presentation and T cell activation. Since ILC2s are permissive for the dengue virus, specifically deleting MHC I or MHC II on ILC2s and studying how that affects the T cell repertoire and function, would delineate capacity of ILC2s to affect T cell priming through antigen presentation.

Together, further studies are needed to shed light on the prospect of ILC2s as additional regulators of T cell-based functions in order to establish long-term protective immunity.

## References

[1] Fan X Rudensky AY. Hallmarks of tissue-resident lymphocytes. Cell. (2016) 164:1198–211. doi: 10.1016/j.cell.2016.02.048, PMID: 26967286 PMC4973889

[2] Schuijs MJ Hammad H Lambrecht BN. Professional and “Amateur” Antigen presenting cells in type 2 immunity. Trends Immunol. (2019) 40:22–34. doi: 10.1016/j.it.2018.11.001, PMID: 30502024 PMC7610811

[3] Gasteiger G Rudensky AY. Opinion: Interactions of innate and adaptive lymphocytes. Nat Rev Immunol. (2014) 14:631–9. doi: 10.1038/nri3726, PMID: 25132095 PMC4504695

[4] Sonnenberg GF Hepworth MR. Functional interactions between innate lymphoid cells and adaptive immunity. Nat Rev Immunol. (2019) 19:599–613. doi: 10.1038/s41577-019-0194-8, PMID: 31350531 PMC6982279

[5] Abramson J Dobeš J Lyu M Sonnenberg GF. The emerging family of RORγt+ antigen-presenting cells. Nat Rev Immunol. (2024) 24:64–77. doi: 10.1038/s41577-023-00906-5, PMID: 37479834 PMC10844842

[6] Eberl G Colonna M Santo JPD McKenzie ANJ. Innate Lymphoid Cells: a new paradigm in immunology. Sci 348 aaa6566. (2015) 348(6237). doi: 10.1126/science.aaa6566, PMID: 25999512 PMC5658207

[7] Gogoi M. ILC2-derived LIF licences progress from tissue to systemic immunity. Nature. (2024) 632:885–92. doi: 10.1038/s41586-024-07746-w, PMID: 39112698 PMC11338826

[8] Jarick KJ. Non-redundant functions of group 2 innate lymphoid cells. Nature. (2022) 611:794–800. doi: 10.1038/s41586-022-05395-5, PMID: 36323785 PMC7614745

[9] Sathe P. Innate immunodeficiency following genetic ablation of Mcl1 in natural killer cells. Nat Commun. (2014) 5:4539. doi: 10.1038/ncomms5539, PMID: 25119382

[10] Szeto ACH. Mef2d potentiates type-2 immune responses and allergic lung inflammation. Science. (2024) 384:eadl0370. doi: 10.1126/science.adl0370, PMID: 38935708 PMC7616247

[11] Tsou AM. Neuropeptide regulation of non-redundant ILC2 responses at barrier surfaces. Nature. (2022) 611:787–93. doi: 10.1038/s41586-022-05297-6, PMID: 36323781 PMC10225046

[12] Zaiss DMW Pearce EJ Artis D McKenzie ANJ Klose CSN. Cooperation of ILC2s and TH2 cells in the expulsion of intestinal helminth parasites. Nat Rev Immunol. (2024) 24:294–302. doi: 10.1038/s41577-023-00942-1, PMID: 37798539

[13] Vivier E. Innate lymphoid cells: 10 years on. Cell. (2018) 174:1054–66. doi: 10.1016/j.cell.2018.07.017, PMID: 30142344

[14] Lopes N Vivier E Narni-Mancinelli E. Natural killer cells and type 1 innate lymphoid cells in cancer. Semin Immunol. (2023) 66:101709. doi: 10.1016/j.smim.2022.101709, PMID: 36621291

[15] Moro K. Innate production of TH2 cytokines by adipose tissue-associated c-Kit+Sca-1+ lymphoid cells. Nature. (2010) 463:540–4. doi: 10.1038/nature08636, PMID: 20023630

[16] Melo-Gonzalez F Hepworth MR. Functional and phenotypic heterogeneity of group 3 innate lymphoid cells. Immunology. (2017) 150:265–75. doi: 10.1111/imm.12697, PMID: 27935637 PMC5290240

[17] Horn V Sonnenberg GF. Group 3 innate lymphoid cells in intestinal health and disease. Nat Rev Gastroenterol Hepatol. (2024) 21:428–43. doi: 10.1038/s41575-024-00906-3, PMID: 38467885 PMC11144103

[18] Nussbaum JC. Type 2 innate lymphoid cells control eosinophil homeostasis. Nature. (2013) 502:245–8. doi: 10.1038/nature12526, PMID: 24037376 PMC3795960

[19] Reichman H. Activated eosinophils exert antitumorigenic activities in colorectal cancer. Cancer Immunol Res. (2019) 7:388–400. doi: 10.1158/2326-6066.CIR-18-0494, PMID: 30665890

[20] Fallon PG. Identification of an interleukin (IL)-25–dependent cell population that provides IL-4, IL-5, and IL-13 at the onset of helminth expulsion. J Exp Med. (2006) 203:1105–16. doi: 10.1084/jem.20051615, PMID: 16606668 PMC2118283

[21] Neill DR. Nuocytes represent a new innate effector leukocyte that mediates type-2 immunity. Nature. (2010) 464:1367–70. doi: 10.1038/nature08900, PMID: 20200518 PMC2862165

[22] Price AE. Systemically dispersed innate IL-13–expressing cells in type 2 immunity. Proc Natl Acad Sci U. S. A. (2010) 107:11489–94. doi: 10.1073/pnas.1003988107, PMID: 20534524 PMC2895098

[23] Huang Y. IL-25-responsive, lineage-negative KLRG1hi cells are multipotential ‘inflammatory’ type 2 innate lymphoid cells. Nat Immunol. (2015) 16:161–9. doi: 10.1038/ni.3078, PMID: 25531830 PMC4297567

[24] Meininger I. Tissue-specific features of innate lymphoid cells. Trends Immunol. (2020) 41:902–17. doi: 10.1016/j.it.2020.08.009, PMID: 32917510

[25] Burrows K. A gut commensal protozoan determines respiratory disease outcomes by shaping pulmonary immunity. Cell. (2025) 188:316–330.e12. doi: 10.1016/j.cell.2024.11.020, PMID: 39706191 PMC11761380

[26] Herbert DR Douglas B Zullo K. Group 2 innate lymphoid cells (ILC2): type 2 immunity and helminth immunity. Int J Mol Sci. (2019) 20:2276. doi: 10.3390/ijms20092276, PMID: 31072011 PMC6539149

[27] Halim TYF Krauss RH Sun AC Takei F. Lung natural helper cells are a critical source of Th2 cell-type cytokines in protease allergen-induced airway inflammation. Immunity. (2012) 36:451–63. doi: 10.1016/j.immuni.2011.12.020, PMID: 22425247

[28] Yudanin NA. Spatial and temporal mapping of human innate lymphoid cells reveals elements of tissue specificity. Immunity. (2019) 50:505–519.e4. doi: 10.1016/j.immuni.2019.01.012, PMID: 30770247 PMC6594374

[29] Halim TYF. Group 2 innate lymphoid cells in disease. Int Immunol. (2016) 28:13–22. doi: 10.1093/intimm/dxv050, PMID: 26306498 PMC5891987

[30] Seillet C Belz GT Mielke LA. Complexity of cytokine network regulation of innate lymphoid cells in protective immunity. Cytokine. (2014) 70:1–10. doi: 10.1016/j.cyto.2014.06.002, PMID: 24972988

[31] Klose CSN Artis D. Innate lymphoid cells as regulators of immunity, inflammation and tissue homeostasis. Nat Immunol. (2016) 17:765–74. doi: 10.1038/ni.3489, PMID: 27328006

[32] Seillet C Jacquelot N. Sensing of physiological regulators by innate lymphoid cells. Cell Mol Immunol. (2019) 16:442–51. doi: 10.1038/s41423-019-0217-1, PMID: 30842626 PMC6474201

[33] Mackley EC. CCR7-dependent trafficking of RORγ+ ILCs creates a unique microenvironment within mucosal draining lymph nodes. Nat Commun. (2015) 6:5862. doi: 10.1038/ncomms6862, PMID: 25575242 PMC4354100

[34] Mathä L. Migration of lung resident group 2 innate lymphoid cells link allergic lung inflammation and liver immunity. Front Immunol. (2021) 12:679509. doi: 10.3389/fimmu.2021.679509, PMID: 34305911 PMC8299566

[35] Kästele V. Intestinal-derived ILCs migrating in lymph increase IFNγ production in response to Salmonella Typhimurium infection. Mucosal Immunol. (2021) 14:717–27. doi: 10.1038/s41385-020-00366-3, PMID: 33414524 PMC8075955

[36] Amisaki M. IL-33-activated ILC2s induce tertiary lymphoid structures in pancreatic cancer. Nature. (2025) 638:1076–84. doi: 10.1038/s41586-024-08426-5, PMID: 39814891 PMC11864983

[37] Huang Y. S1P-dependent interorgan trafficking of group 2 innate lymphoid cells supports host defense. Science. (2018) 359:114–9. doi: 10.1126/science.aam5809, PMID: 29302015 PMC6956613

[38] Chi L. Sexual dimorphism in skin immunity is mediated by an androgen-ILC2-dendritic cell axis. Science. (2024) 384:eadk6200. doi: 10.1126/science.adk6200, PMID: 38574174 PMC12086714

[39] Mayer JU. Homeostatic IL-13 in healthy skin directs dendritic cell differentiation to promote TH2 and inhibit TH17 cell polarization. Nat Immunol. (2021) 22:1538–50. doi: 10.1038/s41590-021-01067-0, PMID: 34795444

[40] Lee SH. Dermis resident macrophages orchestrate localized ILC2 eosinophil circuitries to promote non-healing cutaneous leishmaniasis. Nat Commun. (2023) 14:7852. doi: 10.1038/s41467-023-43588-2, PMID: 38030609 PMC10687111

[41] Ricardo-Gonzalez RR. Innate type 2 immunity controls hair follicle commensalism by. Demodex mites. Immun. (2022) 55:1891–1908.e12., PMID: 36044899 10.1016/j.immuni.2022.08.001 PMC9561030

[42] Kobayashi T Ricardo-Gonzalez RR Moro K. Skin-resident innate lymphoid cells – cutaneous innate guardians and regulators. Trends Immunol. (2020) 41:100–12. doi: 10.1016/j.it.2019.12.004, PMID: 31948873 PMC7364860

[43] Nakatani-Kusakabe M. Monitoring cellular movement with photoconvertible fluorescent protein and single-cell RNA sequencing reveals cutaneous group 2 innate lymphoid cell subtypes, circulating ILC2 and skin-resident ILC2. JID Innov. (2021) 1. doi: 10.1016/j.xjidi.2021.100035, PMID: 34909732 PMC8659747

[44] Dutton EE. Peripheral lymph nodes contain migratory and resident innate lymphoid cell populations. Sci Immunol. (2019) 4:eaau8082. doi: 10.1126/sciimmunol.aau8082, PMID: 31152090 PMC7018521

[45] Imai Y. Skin-specific expression of IL-33 activates group 2 innate lymphoid cells and elicits atopic dermatitis-like inflammation in mice. Proc Natl Acad Sci U. S. A. (2013) 110:13921–6. doi: 10.1073/pnas.1307321110, PMID: 23918359 PMC3752227

[46] Dahlgren MW. Adventitial stromal cells define group 2 innate lymphoid cell tissue niches. Immunity. (2019) 50:707–722.e6. doi: 10.1016/j.immuni.2019.02.002, PMID: 30824323 PMC6553479

[47] Li Y. Kinetics of the accumulation of group 2 innate lymphoid cells in IL-33-induced and IL-25-induced murine models of asthma: a potential role for the chemokine CXCL16. Cell Mol Immunol. (2019) 16:75–86. doi: 10.1038/s41423-018-0182-0, PMID: 30467418 PMC6318283

[48] Wiertsema SP van Bergenhenegouwen J Garssen J Knippels LMJ. The interplay between the gut microbiome and the immune system in the context of infectious diseases throughout life and the role of nutrition in optimizing treatment strategies. Nutrients. (2021) 13:886. doi: 10.3390/nu13030886, PMID: 33803407 PMC8001875

[49] von Moltke J Ji M Liang H-E Locksley RM. Tuft-cell-derived IL-25 regulates an intestinal ILC2–epithelial response circuit. Nature. (2016) 529:221–5. doi: 10.1038/nature16161, PMID: 26675736 PMC4830391

[50] Ricardo-Gonzalez RR. Tissue-specific pathways extrude activated ILC2s to disseminate type 2 immunity. J Exp Med. (2020) 217:e20191172. doi: 10.1084/jem.20191172, PMID: 32031571 PMC7144525

[51] Murphy JM Ngai L Mortha A Crome SQ. Tissue-dependent adaptations and functions of innate lymphoid cells. Front Immunol. (2022) 13:836999. doi: 10.3389/fimmu.2022.836999, PMID: 35359972 PMC8960279

[52] Romera-Hernández M Mathä L Steer CA Ghaedi M Takei F. Identification of group 2 innate lymphoid cells in mouse lung, liver, small intestine, bone marrow, and mediastinal and mesenteric lymph nodes. Curr Protoc Immunol. (2019) 125:e73. doi: 10.1002/cpim.73, PMID: 30994980

[53] Lok LSC. Group 2 innate lymphoid cells exhibit tissue-specific dynamic behaviour during type 2 immune responses. Front Immunol. (2021) 12:711907. doi: 10.3389/fimmu.2021.711907, PMID: 34484215 PMC8415880

[54] Troch KF. Group 2 innate lymphoid cells are a non-redundant source of interleukin-5 required for development and function of murine B1 cells. Nat Commun. (2024) 15:10566. doi: 10.1038/s41467-024-54780-3, PMID: 39632879 PMC11618303

[55] Bresler P. T cells regulate lymph node-resident ILC populations in a tissue and subset-specific way. iScience. (2021) 24:102158. doi: 10.1016/j.isci.2021.102158, PMID: 33665576 PMC7907429

[56] Heul AMV. RAG suppresses group 2 innate lymphoid cells. eLife. (2025) 13. doi: 10.7554/eLife.98287.3 PMC12055012 40326866

[57] Martin CE. Interleukin-7 availability is maintained by a hematopoietic cytokine sink comprising innate lymphoid cells and T cells. Immunity. (2017) 47:171–182.e4. doi: 10.1016/j.immuni.2017.07.005, PMID: 28723549

[58] Wang Y Li D Liu Y Chen S Dong Z. Adaptive immune cells antagonize ILC2 homeostasis via SLAMF3 and SLAMF5. Sci Adv. (2025) 11:eadp9894. doi: 10.1126/sciadv.adp9894, PMID: 39792675 PMC11721569

[59] Kobayashi T. Homeostatic control of sebaceous glands by innate lymphoid cells regulates commensal bacteria equilibrium. Cell. (2019) 176:982–997.e16. doi: 10.1016/j.cell.2018.12.031, PMID: 30712873 PMC6532063

[60] Malhotra N. RORα-expressing T regulatory cells restrain allergic skin inflammation. Sci Immunol. (2018) 3:eaao6923. doi: 10.1126/sciimmunol.aao6923, PMID: 29500225 PMC5912895

[61] Leyva-Castillo JM. ILC2 activation by keratinocyte-derived IL-25 drives IL-13 production at sites of allergic skin inflammation. J Allergy Clin Immunol. (2020) 145:1606–1614.e4. doi: 10.1016/j.jaci.2020.02.026, PMID: 32179159 PMC7282942

[62] Kallal LE Hartigan AJ Hogaboam CM Schaller MA Lukacs NW. Inefficient lymph node sensitization during respiratory viral infection promotes IL-17-mediated lung pathology. J Immunol Baltim. Md 1950. (2010) 185:4137–47. doi: 10.4049/jimmunol.1000677, PMID: 20805422 PMC4417502

[63] Symowski C Voehringer D. Th2 cell-derived IL-4/IL-13 promote ILC2 accumulation in the lung by ILC2-intrinsic STAT6 signaling in mice. Eur J Immunol. (2019) 49:1421–32. doi: 10.1002/eji.201948161, PMID: 31144294

[64] Li BWS. T cells are necessary for ILC2 activation in house dust mite-induced allergic airway inflammation in mice. Eur J Immunol. (2016) 46:1392–403. doi: 10.1002/eji.201546119, PMID: 27062360

[65] Halim TY. Group 2 innate lymphoid cells are critical for the initiation of adaptive T helper 2 cell-mediated allergic lung inflammation. Immunity. (2014) 40:425. doi: 10.1016/j.immuni.2014.01.011, PMID: 24613091 PMC4210641

[66] Schwartz C. ILC2s regulate adaptive Th2 cell functions via PD-L1 checkpoint control. J Exp Med. (2017) 214:2507. doi: 10.1084/jem.20170051, PMID: 28747424 PMC5584124

[67] Maazi H. ICOS: ICOS-Ligand interaction is required for type 2 innate lymphoid cell function, homeostasis and induction of airway hyperreactivity. Immunity. (2015) 42:538–51. doi: 10.1016/j.immuni.2015.02.007, PMID: 25769613 PMC4366271

[68] Muntjewerff EM Meesters LD van den Bogaart G. Antigen cross-presentation by macrophages. Front Immunol. (2020) 11:1276. doi: 10.3389/fimmu.2020.01276, PMID: 32733446 PMC7360722

[69] Rastogi I. Role of B cells as antigen presenting cells. Front Immunol. (2022) 13:954936. doi: 10.3389/fimmu.2022.954936, PMID: 36159874 PMC9493130

[70] Schriek P Villadangos JA. Trogocytosis and cross-dressing in antigen presentation. Curr Opin Immunol. (2023) 83:102331. doi: 10.1016/j.coi.2023.102331, PMID: 37148582

[71] Hardman CS. CD1a presentation of endogenous antigens by group 2 innate lymphoid cells. Sci Immunol. (2017) 2:eaan5918. doi: 10.1126/sciimmunol.aan5918, PMID: 29273672 PMC5826589

[72] Oliphant CJ. MHCII-mediated dialog between group 2 innate lymphoid cells and CD4+ T cells potentiates type 2 immunity and promotes parasitic helminth expulsion. Immunity. (2014) 41:283–95. doi: 10.1016/j.immuni.2014.06.016, PMID: 25088770 PMC4148706

[73] Mirchandani AS. Type 2 innate lymphoid cells drive CD4+ Th2 cell responses. J Immunol. (2014) 192:2442–8. doi: 10.4049/jimmunol.1300974, PMID: 24470502

[74] Hepworth MR. Innate lymphoid cells regulate CD4+ T cell responses to intestinal commensal bacteria. Nature. (2013) 498:113–7. doi: 10.1038/nature12240, PMID: 23698371 PMC3699860

[75] Wen J. Group 2 innate lymphoid cells boost CD8 ^+^ T-cell activation in anti-tumor immune responses. OncoImmunology. (2023) 12:2243112. doi: 10.1080/2162402X.2023.2243112, PMID: 37577145 PMC10413917

[76] Yang Y. Group 2 innate lymphoid cells can engulf and destroy bacteria. Cell Mol Immunol. (2021) 18:2569–71. doi: 10.1038/s41423-021-00765-x, PMID: 34522019 PMC8546081

[77] Kim J. Antigen cross-presentation by type-2 innate lymphoid cells facilitates the activation of anti-tumor CD8+ T cells. Cancer Res. (2025) 85(14):2659–78. doi: 10.1158/0008-5472.CAN-24-4194, PMID: 40245114

[78] Steinmann S. Hepatic ILC2 activity is regulated by liver inflammation-induced cytokines and effector CD4+ T cells. Sci Rep. (2020) 10:1071. doi: 10.1038/s41598-020-57985-w, PMID: 31974518 PMC6978388

[79] Gonzalo JA. ICOS is critical for T helper cell–mediated lung mucosal inflammatory responses. Nat Immunol. (2001) 2:597–604. doi: 10.1038/89739, PMID: 11429543

[80] Watanabe M. Down-regulation of ICOS ligand by interaction with ICOS functions as a regulatory mechanism for immune responses1. J Immunol. (2008) 180:5222–34. doi: 10.4049/jimmunol.180.8.5222, PMID: 18390703

[81] Halim TYF. Tissue-restricted adaptive type 2 immunity is orchestrated by expression of the costimulatory molecule OX40L on group 2 innate lymphoid cells. Immunity. (2018) 48:1195–1207.e6. doi: 10.1016/j.immuni.2018.05.003, PMID: 29907525 PMC6015114

[82] Stockis J. Cross-talk between ILC2 and Gata3high Tregs locally constrains adaptive type 2 immunity. Sci Immunol. (2024) 9:eadl1903. doi: 10.1126/sciimmunol.adl1903, PMID: 39028828

[83] Frech M. Btn2a2 regulates ILC2–T cell cross talk in type 2 immune responses. Front Immunol. (2022) 13:757436. doi: 10.3389/fimmu.2022.757436, PMID: 35145516 PMC8821520

[84] Drake LY Iijima K Kita H. Group 2 innate lymphoid cells and CD4+ T cells cooperate to mediate type 2 immune response in mice. Allergy. (2014) 69:1300–7. doi: 10.1111/all.12446, PMID: 24939388 PMC4160406

[85] Wan J. ILC2-derived IL-9 inhibits colorectal cancer progression by activating CD8+ T cells. Cancer Lett. (2021) 502:34–43. doi: 10.1016/j.canlet.2021.01.002, PMID: 33429004

[86] Wilhelm C. An IL-9 fate reporter demonstrates the induction of an innate IL-9 response in lung inflammation. Nat Immunol. (2011) 12:1071–7. doi: 10.1038/ni.2133, PMID: 21983833 PMC3198843

[87] Duerr CU. Type I interferon restricts type 2 immunopathology through the regulation of group 2 innate lymphoid cells. Nat Immunol. (2016) 17:65–75. doi: 10.1038/ni.3308, PMID: 26595887 PMC9135352

[88] Monticelli LA. IL-33 promotes an innate immune pathway of intestinal tissue protection dependent on amphiregulin–EGFR interactions. Proc Natl Acad Sci. (2015) 112:10762–7. doi: 10.1073/pnas.1509070112, PMID: 26243875 PMC4553775

[89] Minutti CM. A macrophage-pericyte axis directs tissue restoration via amphiregulin-induced transforming growth factor beta activation. Immunity. (2019) 50:645–654.e6. doi: 10.1016/j.immuni.2019.01.008, PMID: 30770250 PMC6436929

[90] Kelly A. Human monocytes and macrophages regulate immune tolerance via integrin αvβ8–mediated TGFβ activation. J Exp Med. (2018) 215:2725–36. doi: 10.1084/jem.20171491, PMID: 30355614 PMC6219736

[91] Wachtendorf S. The ST2+ Treg/amphiregulin axis protects from immune-mediated hepatitis. Front Immunol. (2024) 15. doi: 10.3389/fimmu.2024.1351405, PMID: 38571949 PMC10987816

[92] Wang S. Amphiregulin confers regulatory T cell suppressive function and tumor invasion via the EGFR/GSK-3β/foxp3 axis. J Biol Chem. (2016) 291:21085–95. doi: 10.1074/jbc.M116.717892, PMID: 27432879 PMC5076518

[93] Zaiss DMW. Amphiregulin enhances regulatory T cell suppressive function via the epidermal growth factor receptor. Immunity. (2013) 38:275–84. doi: 10.1016/j.immuni.2012.09.023, PMID: 23333074 PMC3582723

[94] Ryu S. The protective roles of integrin α4β7 and Amphiregulin-expressing innate lymphoid cells in lupus nephritis. Cell Mol Immunol. (2024) 21:723–37. doi: 10.1038/s41423-024-01178-2, PMID: 38806623 PMC11214630

[95] Zaiss DMW Gause WC Osborne LC Artis D. Emerging functions of amphiregulin in orchestrating immunity, inflammation and tissue repair. Immunity. (2015) 42:216–26. doi: 10.1016/j.immuni.2015.01.020, PMID: 25692699 PMC4792035

[96] Halim TY. Group 2 innate lymphoid cells license dendritic cells to potentiate memory T helper 2 cell responses. Nat Immunol. (2016) 17:57–64. doi: 10.1038/ni.3294, PMID: 26523868 PMC4685755

[97] Mortha A Burrows K. Cytokine networks between innate lymphoid cells and myeloid cells. Front Immunol. (2018) 9. doi: 10.3389/fimmu.2018.00191, PMID: 29467768 PMC5808287

[98] Chevalier MF. ILC2-modulated T cell–to-MDSC balance is associated with bladder cancer recurrence. J Clin Invest. (2017) 127:2916–29. doi: 10.1172/JCI89717, PMID: 28650339 PMC5531411

[99] Grisaru-Tal S Rothenberg ME Munitz A. Eosinophil–lymphocyte interactions in the tumor microenvironment and cancer immunotherapy. Nat Immunol. (2022) 23:1309–16. doi: 10.1038/s41590-022-01291-2, PMID: 36002647 PMC9554620

[100] Wang S. Transdifferentiation of tumor infiltrating innate lymphoid cells during progression of colorectal cancer. Cell Res. (2020) 30:610–22. doi: 10.1038/s41422-020-0312-y, PMID: 32367039 PMC7343789

[101] Moral JA. ILC2s amplify PD-1 blockade by activating tissue-specific cancer immunity. Nature. (2020) 579:130–5. doi: 10.1038/s41586-020-2015-4, PMID: 32076273 PMC7060130

[102] Jacquelot N Seillet C Vivier E Belz GT. Innate lymphoid cells and cancer. Nat Immunol. (2022) 23:371–9. doi: 10.1038/s41590-022-01127-z, PMID: 35228695

[103] Nurieva RI. Bcl6 mediates the development of T follicular helper cells. Science. (2009) 325:1001–5. doi: 10.1126/science.1176676, PMID: 19628815 PMC2857334

[104] Arkatkar T. B cell–derived IL-6 initiates spontaneous germinal center formation during systemic autoimmunity. J Exp Med. (2017) 214:3207–17. doi: 10.1084/jem.20170580, PMID: 28899868 PMC5679179

[105] Dudeck J. Mast cells acquire MHCII from dendritic cells during skin inflammation. J Exp Med. (2017) 214:3791–811. doi: 10.1084/jem.20160783, PMID: 29084819 PMC5716026

[106] Fonseka CL. Dengue virus co-opts innate type 2 pathways to escape early control of viral replication. Commun Biol. (2022) 5:735. doi: 10.1038/s42003-022-03682-5, PMID: 35869167 PMC9306424

